# Systemic inflammation response index as an independent predictor of unfavorable prognosis and its application in risk stratification in patients with aneurysmal subarachnoid hemorrhage

**DOI:** 10.3389/fnins.2026.1817586

**Published:** 2026-05-20

**Authors:** Ming-Pei Zhao, Xiao-Dan Zhang, Neng Wang, Chen-Yang Huang, Yi-Xiao Li, De-Zhi Kang, Zong-Qing Zheng, Yuan-Xiang Lin

**Affiliations:** 1 Department of Neurosurgery, Neurosurgery Research Institute, The First Affiliated Hospital, Fujian Medical University, Fuzhou, Fujian, China; 2 Department of Neurosurgery, National Regional Medical Center, Binhai Campus of the First Affiliated Hospital, Fujian Medical University, Fuzhou, Fujian, China; 3 Fujian Provincial Institutes of Brain Disorders and Brain Sciences, First Affiliated Hospital, Fujian Medical University, Fuzhou, Fujian, China; 4 Fujian Provincial Clinical Research Center for Neurological Diseases, First Affiliated Hospital, Fujian Medical University, Fuzhou, Fujian, China; 5 Clinical Research and Translation Center, The First Affiliated Hospital, Fujian Medical University, Fuzhou, Fujian, China; 6 The School of Basic Medical Sciences, Fujian Medical University, Fuzhou, Fujian, China

**Keywords:** aneurysmal subarachnoid hemorrhage (aSAH), inflammatory biomarker, nomogram, risk stratification, stroke, systemic inflammation response index (SIRI), unfavorable prognosis

## Abstract

**Background:**

Aneurysmal subarachnoid hemorrhage (aSAH) is a devastating cerebrovascular disease associated with high rates of mortality and long-term disability. Early risk stratification is essential to guide personalized management. Systemic inflammation plays a key role in secondary brain injury after aSAH. The systemic inflammation response index (SIRI), a novel inflammatory marker combining neutrophil, monocyte, and lymphocyte counts, has shown prognostic value in multiple disorders, but its long-term prognostic role in aSAH remains unclear.

**Objectives:**

This study aimed to investigate the association between admission SIRI and 12-month unfavorable functional outcomes (modified Rankin Scale [mRS] ≥ 3) in patients with aSAH, verify its independent prognostic value, and construct a clinically useful prediction nomogram.

**Methods:**

A retrospective cohort study was performed including 258 patients with aSAH admitted between January 2021 and December 2024. Patients were divided into a favorable prognosis group (mRS 0–2, *n =* 158) and an unfavorable prognosis group (mRS ≥ 3, *n =* 100). Baseline characteristics, imaging indices including modified Fisher scale, laboratory parameters, and treatment data were collected. Multivariate logistic regression with forced entry was used to identify independent prognostic factors. Restricted cubic spline (RCS) analysis was applied to explore the non-linear relationship between SIRI and prognosis. A prediction nomogram was constructed and validated using temporal validation (training cohort *n =* 170; validation cohort *n =* 88). Model performance was evaluated using discrimination, calibration, and decision curve analysis.

**Results:**

SIRI was significantly higher in the unfavorable prognosis group (*p <* 0.001). Multivariate analysis confirmed that SIRI (OR = 1.20, 95% CI: 1.08–1.34, *p =* 0.001), age, hypertension, GCS score ≤ 8, modified Fisher scale, and treatment modality were independent prognostic factors. RCS analysis demonstrated a non-linear relationship (P for nonlinearity = 0.020), with a clear threshold at SIRI = 4.36; the risk of unfavorable outcomes rose steeply above this cutoff. The nomogram showed excellent discrimination (AUC = 0.881 in training; 0.919 in validation) and satisfactory calibration. Decision curve analysis confirmed favorable clinical utility.

**Conclusion:**

Admission SIRI is an independent predictor of 12-month unfavorable functional outcomes in patients with aSAH. A threshold value of 4.36 can effectively identify high-risk patients. The SIRI-integrated nomogram provides accurate and individualized prognosis prediction across both training and temporal validation cohorts. This validated tool provides robust evidence to support clinical risk stratification and personalized decision-making.

## Introduction

Spontaneous aneurysmal subarachnoid hemorrhage (aSAH) stands as one of the most devastating subtypes of stroke (Suarez et al., 2006). Accounting for roughly 5–10% of all cerebrovascular events globally, it differs distinctly from ischemic stroke in its clinical trajectory: onset is abrupt, neurological damage progresses rapidly, and long-term outcomes remain dismal. During the acute phase, mortality rates range from 35 to 40%, and nearly half of survivors are left with moderate to severe neurological disability 6 months post-onset (Suarez et al., 2006; Macdonald and Schweizer, 2017; Natasha et al., 2019). Such outcomes not only rob patients of their ability to manage daily activities independently but also place heavy socioeconomic strains on families and healthcare systems alike.

Early identification of patients at high risk of unfavorable outcomes is pivotal to optimizing clinical management of aSAH (Rohan et al., 2024). Currently, prognostic assessment in clinical practice primarily relies on disease severity scales (e.g., Hunt–Hess grade and Glasgow Coma Scale [GCS] score), imaging characteristics (aneurysm size, location, and intraventricular hemorrhage), and baseline comorbidities (hypertension and diabetes mellitus) (Vivien et al., 2014; Vesna et al., 2022; Rohan et al., 2024). However, these conventional markers carry inherent limitations: they overlook the dynamic pathophysiological cascades driving aSAH progression—most notably the pivotal role of systemic inflammation in mediating secondary brain injury and shaping long-term functional recovery.

Aneurysm rupture triggers a robust systemic inflammatory response that lies at the heart of the pathophysiological cascade in aSAH (Michal et al., 2019; Jing et al., 2022). Following the leakage of blood into the subarachnoid space, erythrocyte lysis releases hemoglobin and heme, two key molecules that trigger complement system activation and engage multiple inflammation-associated signaling pathways (Rijin et al., 2025). Subsequent to this pro-inflammatory cascade initiation, pro-inflammatory cells—predominantly neutrophils and monocytes—are recruited to the brain injury locus (Axel et al., 2019; Bingrui et al., 2025). At this site, these infiltrating cells secrete reactive oxygen species and pro-inflammatory cytokines such as interleukin-6 and tumor necrosis factor-*α*, further aggravating blood–brain barrier impairment and cerebral vasospasm (Jing et al., 2022). Meanwhile, lymphocyte suppression takes hold, upsetting the balance between pro-inflammatory and anti-inflammatory responses and hindering neurorepair mechanisms (Jing et al., 2022; Xie and Enqiang, 2022). This inflammatory dysregulation drives poor functional outcomes, underscoring the need for a quantifiable marker to assess systemic inflammation in patients with aSAH.

In recent years, peripheral blood-based inflammatory indices have emerged as promising prognostic tools across various diseases (Jinming et al., 2020; Fenfang et al., 2025). Their appeal stems from their ready accessibility, low cost, and ability to reflect the balance between systemic inflammation and immune homeostasis. Among these indices, the systemic inflammation response index (SIRI)—calculated as (neutrophil count × monocyte count)/lymphocyte count—has gained growing attention for a distinct advantage: it synthesizes dynamic changes in both pro-inflammatory cells (neutrophils and monocytes) and anti-inflammatory cells (lymphocytes) (Liu et al., 2016; Linguo et al., 2024; Fei-Ran et al., 2025). This integration enables it to offer a more holistic assessment of systemic inflammatory status than single-cell indices (e.g., white blood cell count) or simple ratios (e.g., neutrophil-to-lymphocyte ratio (NLR)).

To date, SIRI has proven its worth as a reliable prognostic marker across multiple clinical settings. In acute ischemic stroke, higher SIRI levels are tied to an elevated risk of unfavorable 3-month outcomes and mortality (Song et al., 2025); in severe pneumonia, it forecasts in-hospital death and progression to respiratory failure (Cosmin et al., 2022); and in solid tumors like colorectal and lung cancer, it correlates with tumor stage and long-term survival (Herong and Wei, 2024; Otilia et al., 2024). Notably, in intracerebral hemorrhage—another hemorrhagic cerebrovascular disorder that shares similar inflammatory pathophysiology with aSAH—the SIRI has been shown to independently predict hematoma expansion and poor 90-day functional outcomes (Ziyi et al., 2025). A previous single-center study (Zhaobo et al., 2023) identified the SIRI as an independent predictor of 90-day short-term outcomes in aSAH patients, but it focused only on short-term prognosis and did not explore SIRI’s non-linear relationship with outcomes or develop a practical clinical tool for risk stratification. Given these gaps and the critical need for reliable long-term prognostic markers to guide individualized care, the present study aimed to investigate the association between admission SIRI and unfavorable outcomes in aSAH patients, validate its independent prognostic value, and explore its clinical utility for risk stratification.

This study had four key objectives: first, to explore the link between admission SIRI and unfavorable 12-month outcomes (modified Rankin Scale [mRS] score ≥3) in patients with aSAH; second, to confirm whether the SIRI serves as an independent prognostic factor for aSAH through multivariate regression analysis; third, to characterize the non-linear dose–response relationship between the SIRI and prognostic risk via restricted cubic spline (RCS) analysis; and fourth, to develop a visualized nomogram integrated with the SIRI, offering a practical tool for clinical risk stratification. The findings of this study further reinforce the role of inflammatory markers in the prognostic evaluation of aSAH and thereby inform individualized clinical management strategies.

## Materials and methods

### Study design and population

This retrospective observational study was based on data extracted from the electronic medical record (EMR) system of the First Affiliated Hospital of Fujian Medical University. The study cohort included patients with aneurysmal subarachnoid hemorrhage (aSAH) admitted to the Department of Neurosurgery between January 2021 and December 2024. This study adhered to the RECORD statement and the Declaration of Helsinki and was approved by the Ethics Committee of the First Affiliated Hospital of Fujian Medical University. Owing to the retrospective design, the requirement for written informed consent was waived by the ethics committee.

Patients were eligible for inclusion if they satisfied the following criteria: confirmed diagnosis of aSAH via digital subtraction angiography (DSA) or computed tomography angiography (CTA); age ≥18 years; admitted to the hospital within 72 h of symptom onset and had completed baseline clinical, laboratory, and imaging data (including modified Fisher grade) collection; and a minimum 12-month follow-up after discharge with a valid mRS score at the 12-month assessment.

Patients were excluded if they presented with traumatic subarachnoid hemorrhage; subarachnoid hemorrhage stemming from non-aneurysmal causes (e.g., arteriovenous malformations, brain tumors, or coagulation disorders); pre-admission diagnoses of malignant tumors, end-stage liver or kidney disease, or autoimmune conditions (e.g., rheumatoid arthritis or systemic lupus erythematosus); acute infections (e.g., pneumonia or urinary tract infection) within 1 week before admission; surgical intervention conducted before baseline laboratory testing; or incomplete follow-up data or loss to follow-up.

### Definition of variables

#### Outcome variable

The primary outcome was 12-month functional prognosis, evaluated using the mRS. This seven-point scale ranges from zero (no symptoms) to six (death), with patients stratified into two groups: the favorable prognosis group (mRS 0–2), defined as patients who can perform daily activities independently without or with mild disability, and the unfavorable prognosis group (mRS ≥ 3), characterized by the inability to manage daily activities independently due to moderate to severe disability or death. mRS scores were assessed by two trained neurosurgeons blinded to patients’ baseline data; any discrepancies were resolved through consensus with a third senior neurologist.

#### Exposure variable

The primary exposure variable was the SIRI, computed as (neutrophil count [×10^9^/L] × monocyte count [×10^9^/L]) / lymphocyte count [×10^9^/L]. Peripheral venous blood sample data were retrieved from the hospital’s electronic medical record system, with samples collected within 24 h (median: 9.6 h, IQR: 4.3–17.5 h) of admission—before any invasive procedures or medications that might alter laboratory results. The hospital’s clinical laboratory analyzed all samples as part of routine clinical testing.

#### Covariates

All the following covariates were included in the statistical models to adjust for potential confounding effects on both the exposure variable SIRI and the outcome variable:

- *Demographic characteristics*: Age and gender.
- *Comorbidities*: Hypertension (defined by a pre-existing physician diagnosis, pre-admission use of antihypertensive medications, or admission systolic blood pressure [SBP] ≥ 140 mmHg and/or diastolic blood pressure [DBP] ≥ 90 mmHg), diabetes mellitus (established via a prior physician diagnosis, pre-admission antidiabetic drug or insulin use, or fasting blood glucose ≥7.0 mmol/L), smoking history (≥ one cigarette/day for over 1 year), and alcohol consumption (≥3 episodes/week for over 1 year).
- *Vital signs*: Admission temperature, SBP, and DBP—all measured within 2 h of hospital admission per standard clinical practice.
- *Clinical severity scores*: Hunt–Hess grade and GCS score (strictly reflecting the initial pre-intubation and pre-sedation neurological status within 2 h of admission).
- *Imaging indicators*: Maximum aneurysm diameter, parent artery distribution (stratified as anterior cerebral artery [ACA], anterior communicating artery [ACom], internal carotid artery [ICA], middle cerebral artery [MCA], posterior communicating artery [PCom], or other arterial territories), and modified Fisher grade.
- *Laboratory indicators*: White blood cell count (WBC), neutrophil count (NEUT), lymphocyte count (LYMPH), monocyte count (MONO), hemoglobin (HGB), platelet count (PLT), international normalized ratio (INR), activated partial thromboplastin time (APTT), albumin (ALB), and serum potassium (K).
- *Treatment modality*: Medical management (including bed rest, vital sign monitoring, blood pressure control, nimodipine-mediated cerebral vasospasm prevention, and intracranial pressure reduction), endovascular therapy (endovascular coiling, with stent-assisted coiling included), and microsurgical clipping (direct surgical clipping of the aneurysm neck).

### Definition of complications

Four major postoperative and in-hospital complications were documented and defined according to standard diagnostic criteria for aneurysmal subarachnoid hemorrhage: delayed cerebral ischemia (DCI), cerebral vasospasm, hydrocephalus, and intracranial rebleeding.

DCI was defined as the occurrence of new neurological deterioration or cerebral infarction confirmed on follow-up computed tomography, excluding other identifiable causes. Cerebral vasospasm was diagnosed based on transcranial Doppler ultrasonography elevation of blood flow velocity or angiographic narrowing of intracranial arteries combined with corresponding clinical manifestations. Hydrocephalus was defined as ventricular enlargement on cranial imaging accompanied by clinical signs of intracranial hypertension requiring therapeutic intervention. Intracranial rebleeding was confirmed by repeated cranial computed tomography showing new or recurrent subarachnoid or intracerebral hemorrhage during hospitalization.

### Imaging and laboratory analysis

Baseline imaging data (CTA or DSA) underwent review by two senior neuroradiologists with over a decade of clinical experience, both blinded to patients’ prognostic outcomes to mitigate bias. Aneurysm maximum diameter was quantified on the axial CTA/DSA slice capturing the lesion’s most prominent cross-section, with inter-observer consistency evaluated via the intraclass correlation coefficient—a statistical metric ideal for assessing agreement between multiple raters in quantitative measurements. The modified Fisher grade was assessed according to standard criteria to evaluate the amount and distribution of subarachnoid hemorrhage. Hunt–Hess grading aligned with standard classification criteria, determined by integrating the patient’s consciousness level at admission, presence of focal neurological deficits, and headache severity. Trained neurosurgeons administered the 15-point GCS score within 2 h of admission, evaluating eye opening, verbal response, and motor function; intra-rater reliability was corroborated through repeated assessments to ensure measurement stability.

Laboratory specimens were processed within 24 h of collection to preserve sample integrity. Complete blood count parameters (WBC, NEUT, LYMPH, MONO, HGB, and PLT), coagulation function indices (INR and APTT), and biochemical markers (ALB and K) were analyzed according to the hospital’s routine clinical laboratory protocols. Daily quality control procedures were implemented using standardized reference materials, safeguarding the accuracy and reproducibility of all laboratory measurements.

### Follow-up

All patients completed a 12-month post-discharge follow-up period, consistent with the standard timeframe recommended for aSAH prognostic evaluation. Follow-up was tailored to patient circumstances: the vast majority of patients (*n =* 233, 90.3%) completed in-person outpatient visits for comprehensive neurological examination and functional assessment, while a small subset (*n =* 25, 9.7%) were evaluated via remote methods (telephone interviews or video consultations) for those unable to attend clinic appointments. To ensure assessment consistency, all remote mRS evaluations were conducted by trained clinical researchers using a structured interview format, which has been widely validated in cerebrovascular research to yield high reliability comparable to in-person assessments (Wilson et al., 2002; Bruno et al., 2011). During these interactions, researchers documented key outcomes, including functional status (assessed via the mRS), readmission details (with specific documentation of underlying causes), and mortality status. Patients were classified as lost to follow-up if no contact was established for more than 3 months despite at least three attempts using distinct communication channels. These cases were excluded from the final analysis to limit potential bias.

### Statistical analysis

A total of 258 patients diagnosed with aSAH were enrolled, with clinical records and laboratory test results analyzed comprehensively. Statistical analyses were conducted using three software platforms: SPSS 23.0 (IBM, USA), Stata 17.0 (StataCorp, USA), and R (version 4.3.2; R Foundation for Statistical Computing, Austria). Normality of continuous variables was assessed using the Shapiro–Wilk test, and homoscedasticity was verified using the Levene test.

Patients were grouped based on 12-month post-discharge functional outcomes, measured using the mRS: favorable (mRS 0–2) and unfavorable (mRS ≥ 3). Normally distributed continuous variables are reported as the mean ± standard deviation (x̄±s), with intergroup comparisons conducted using the independent samples *t*-test. Non-normally distributed continuous variables are presented as the median (interquartile range) [M (Q1, Q3)], and group differences were analyzed via the Mann–Whitney U test. Categorical variables are reported as frequencies (percentages) [*n* (%)], with group comparisons performed using the χ^2^ test or Fisher’s exact test, as appropriate.

Patients were divided into a training cohort (2021–2023, *n =* 170) and a temporal validation cohort (2024, *n =* 88) according to admission date. Baseline characteristics were described in the total cohort, and the prediction model was developed in the training cohort.

Multivariate logistic regression was performed using a forced-entry method to identify independent prognostic factors. Considering the number of unfavorable outcome events (*n =* 71) in the training cohort, candidate variables were restricted to ensure model stability and minimize overfitting.

Based on clinical importance and established prognostic relevance, seven variables were predefined and forced into the model: age, hypertension, diabetes mellitus, GCS ≤ 8, modified Fisher scale, SIRI, and treatment method. According to previous clinical studies, GCS score was prioritized over Hunt–Hess grade for evaluating neurological impairment, and modified Fisher scale was preferred over maximum aneurysm diameter for assessing hemorrhage burden. To avoid multicollinearity and redundant adjustment, Hunt–Hess grade was excluded due to high collinearity with GCS score, and maximum aneurysm diameter was omitted because it closely reflects bleeding severity captured by modified Fisher scale. This *a priori* predefined variable selection ensured clear and independent interpretation of neurological dysfunction and hemorrhagic injury, while maintaining an appropriate event-to-variable ratio to reduce overfitting risk.

A prognostic nomogram was constructed using the R rms package based on the multivariate model in the training cohort.

Model performance was validated in the independent temporal validation cohort using three metrics:

1. Discrimination: The area under the ROC curve (AUC) with 95% CI;
2. Calibration: Calibration curves and the Hosmer–Lemeshow goodness-of-fit test;
3. Clinical utility: Decision curve analysis (DCA) to assess net clinical benefit across threshold probabilities.

Restricted cubic spline (RCS) analysis was performed in the training cohort to explore the non-linear dose–response relationship between SIRI and unfavorable outcomes. The optimal cutoff value of SIRI was determined using the Youden index.

A two-sided *p <* 0.05 was considered statistically significant.

## Results

### Baseline characteristics of the study population

**Figure 1:**
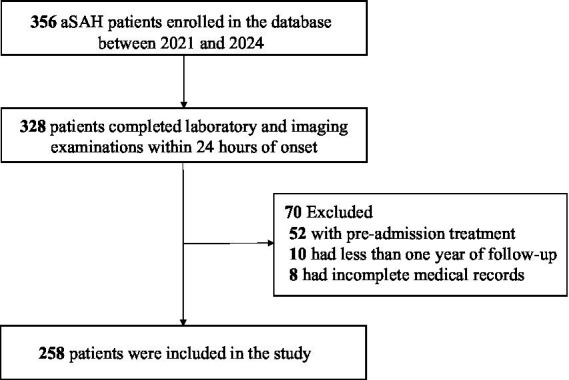
Flowchart of study patients.

A total of 258 patients with aSAH were included in the final analysis, with the detailed patient selection workflow illustrated in Figure 1. Key demographic, clinical, laboratory, and radiological characteristics of the study population are summarized in Table 1. The mean age of the overall cohort was 59.67 ± 11.99 years; 169 patients (65.5%) were female and 89 (34.5%) were male. Hypertension was the most prevalent comorbidity, affecting 138 patients (53.5%), while diabetes mellitus was present in 20 patients (7.8%).

**Table 1:** Comparison of baseline clinical, laboratory, and radiological characteristics according to the modified Rankin Scale groups.

Variable	Overall (*n =* 258)	mRS < 3 (*n =* 158)	mRS ≥ 3 (*n =* 100)	*p*-value
Age, y, mean ± SD	59.67 ± 11.99	58.22 ± 10.94	61.97 ± 13.21	0.0187
Sex, *n* (%)	Female: 169 (65.5%)	Female: 101 (63.9%)	Female: 68 (68.0%)	0.5915
	Male: 89 (34.5%)	Male: 57 (36.1%)	Male: 32 (32.0%)	
Hypertension, *n* (%)	138 (53.5%)	72 (45.6%)	66 (66.0%)	0.0021
Diabetes mellitus, *n* (%)	20 (7.8%)	9 (5.7%)	11 (11.0%)	0.1891
Smoking, *n* (%)	17 (6.6%)	11 (7.0%)	6 (6.0%)	0.9634
Alcohol use, *n* (%)	8 (3.1%)	6 (3.8%)	2 (2.0%)	0.4902
Temperature, °C, mean ± SD	36.70 ± 0.38	36.69 ± 0.28	36.71 ± 0.50	0.7313
Admission SBP, mmHg, mean ± SD	145.29 ± 24.07	143.33 ± 22.34	148.39 ± 26.40	0.1135
Admission DBP, mmHg, mean ± SD	83.74 ± 15.06	82.61 ± 13.01	85.53 ± 17.77	0.1579
GCS ≤ 8, *n* (%)	56 (21.7%)	9 (5.7%)	47 (47.0%)	<0.001
Hunt-Hess>2, *n* (%)	124 (48.1%)	43 (27.2%)	81 (81.0%)	<0.001
Maximum diameter, mm, mean ± SD	6.17 ± 4.48	5.62 ± 3.42	7.04 ± 5.68	0.026
WBC, 10⁹/L, mean ± SD	12.01 ± 4.52	10.66 ± 3.50	14.14 ± 5.11	<0.001
NEUT, 10⁹/L, mean ± SD	10.32 ± 4.47	8.94 ± 3.47	12.52 ± 4.98	<0.001
LYMPH, 10⁹/L, mean ± SD	1.21 ± 0.94	1.33 ± 1.10	1.02 ± 0.55	0.0028
MONO, 10⁹/L, mean ± SD	0.46 ± 0.30	0.39 ± 0.26	0.57 ± 0.31	<0.001
HGB, g/L, mean ± SD	125.72 ± 18.27	127.22 ± 16.68	123.34 ± 20.40	0.1124
PLT, 10⁹/L, mean ± SD	217.10 ± 67.90	221.53 ± 64.91	210.10 ± 72.15	0.1991
ALB, g/L, mean ± SD	41.01 ± 4.66	41.43 ± 3.93	40.35 ± 5.59	0.0939
K, mmol/L, mean ± SD	3.92 ± 0.55	3.94 ± 0.56	3.89 ± 0.53	0.5323
NA, mmol/L, mean ± SD	139.65 ± 13.04	140.16 ± 4.33	138.85 ± 20.26	0.5256
INR, mean ± SD	1.03 ± 0.11	1.02 ± 0.09	1.06 ± 0.12	0.0033
APTT, s, mean ± SD	28.16 ± 6.66	27.51 ± 5.97	29.18 ± 7.54	0.063
NLR, mean ± SD	11.64 ± 8.61	9.07 ± 6.01	15.71 ± 10.38	<0.001
PLR, mean ± SD	232.39 ± 131.51	216.37 ± 120.26	257.70 ± 144.60	0.0182
LMR, mean ± SD	4.58 ± 11.88	6.00 ± 14.96	2.32 ± 1.74	0.0026
SII, 10⁹/L, mean ± SD	2559.40 ± 2131.78	2063.25 ± 1668.65	3343.32 ± 2523.50	<0.001
SIRI, 10⁹/L, mean ± SD	5.65 ± 6.96	3.29 ± 3.47	9.37 ± 9.16	<0.001
Treatment method, *n* (%)	Med: 27 (10.5%)	Med: 6 (3.8%)	Med: 21 (21.0%)	<0.001
	Endo: 130 (50.4%)	Endo: 92 (58.2%)	Endo: 38 (38.0%)	
	Mic: 101 (39.1%)	Mic: 60 (38.0%)	Mic: 41 (41.0%)	
Parent Artery, *n* (%)	ACA: 16 (6.2%)	ACA: 10 (6.3%)	ACA: 6 (6.0%)	0.0457
	ACom: 46 (17.8%)	ACom: 34 (21.5%)	ACom: 12 (12.0%)	
	ICA: 67 (26.0%)	ICA: 31 (19.6%)	ICA: 36 (36.0%)	
	MCA: 40 (15.5%)	MCA: 23 (14.6%)	MCA: 17 (17.0%)	
	PCom: 46 (17.8%)	PCom: 32 (20.3%)	PCom: 14 (14.0%)	
	Other: 43 (16.7%)	Other: 28 (17.7%)	Other: 15 (15.0%)	

SD, Standard Deviation; mRS, Modified Rankin Scale; SBP, Systolic Blood Pressure; DBP, Diastolic Blood Pressure; GCS, Glasgow Coma Scale; Hunt-Hess, Hunt-Hess Scale; WBC, White Blood Cell Count; NEUT, Neutrophil Count; LYMPH, Lymphocyte Count; MONO, Monocyte Count; HGB, Hemoglobin; PLT, Platelet Count; ALB, Albumin; K, Kalium (Potassium); NA, Natrium; INR, International Normalized Ratio; APTT, Activated Partial Thromboplastin Time; NLR, Neutrophil-to-Lymphocyte Ratio; PLR, Platelet-to-Lymphocyte Ratio; LMR, Lymphocyte-to-Monocyte Ratio; SII, Systemic Immune-Inflammation Index; SIRI, Systemic Inflammation Response Index; Endo, Endovascular Therapy; Med, Medical Management; Mic, Microsurgical Clipping; ACA, Anterior Cerebral Artery; ACom, Anterior Communicating Artery; ICA, Internal Carotid Artery; MCA, Middle Cerebral Artery; PCom, Posterior Communicating Artery.

According to 12-month functional outcomes, 158 patients (61.2%) were classified into the favorable prognosis group (mRS 0–2) and 100 patients (38.8%) into the unfavorable prognosis group (mRS ≥ 3). Between-group comparisons revealed significant differences in multiple baseline characteristics.

Patients in the unfavorable outcome group were significantly older (61.97 ± 13.21 vs. 58.22 ± 10.94 years, *p =* 0.019) and more frequently had hypertension (66.0% vs. 45.6%, *p =* 0.002). The maximum aneurysm diameter was significantly larger in the unfavorable outcome group (7.04 ± 5.68 vs. 5.62 ± 3.42 mm, *p =* 0.026).

Marked intergroup differences were observed in admission disease severity. The unfavorable outcome group had a substantially higher proportion of patients with GCS score ≤ 8 (47.0% vs. 5.7%, *p <* 0.001), Hunt–Hess grade > 2 (81.0% vs. 27.2%, *p <* 0.001), and higher modified Fisher grade (median [IQR]: 3 [2–4] vs. 2 [2–3], *p <* 0.001).

For laboratory parameters, patients with unfavorable outcomes exhibited significantly higher levels of WBC (14.14 ± 5.11 vs. 10.66 ± 3.50 × 10^9^/L, *p <* 0.001), NEUT (12.52 ± 4.98 vs. 8.94 ± 3.47 × 10^9^/L, *p <* 0.001), and MONO (0.57 ± 0.31 vs. 0.39 ± 0.26 × 10^9^/L, *p <* 0.001). Inflammatory indices including NLR (15.71 ± 10.38 vs. 9.07 ± 6.01, *p <* 0.001), PLR (257.70 ± 144.60 vs. 216.37 ± 120.26, *p =* 0.018), SII (3343.32 ± 2523.50 vs. 2063.25 ± 1668.65, *p <* 0.001), and SIRI (9.37 ± 9.16 vs. 3.29 ± 3.47, *p <* 0.001) were all significantly elevated in the unfavorable outcome group. In contrast, LYMPH (1.02 ± 0.55 vs. 1.33 ± 1.10 × 10^9^/L, *p =* 0.003) and LMR (2.32 ± 1.74 vs. 6.00 ± 14.96, *p =* 0.003) were significantly lower in this group. The INR was also higher in patients with unfavorable outcomes (1.06 ± 0.12 vs. 1.02 ± 0.09, *p =* 0.003).

Treatment modality differed significantly between groups (*p <* 0.001). Patients with unfavorable outcomes more often received conservative medical management alone (21.0% vs. 3.8%) and less often underwent endovascular therapy (38.0% vs. 58.2%). The distribution of parent arteries also differed significantly between the two groups (*p =* 0.046).

For in-hospital complications, the unfavorable outcome group had significantly higher rates of delayed cerebral ischemia (DCI) (25.0% vs. 13.3%, *p =* 0.022), cerebral vasospasm (32.0% vs. 16.5%, *p =* 0.005), and intracranial rebleeding (20.0% vs. 7.6%, *p =* 0.003). The incidence of hydrocephalus was comparable between groups (*p =* 0.450).

These baseline findings demonstrate that older age, hypertension, larger aneurysm size, more severe admission neurological status, elevated systemic inflammatory markers (including SIRI), conservative treatment, and major postoperative complications are closely associated with unfavorable 12-month functional outcomes in patients with aSAH, providing a basis for subsequent multivariate prognostic analysis. Furthermore, baseline demographics, clinical features, and laboratory parameters were well-balanced between the training cohort (*n =* 170) and the temporal validation cohort (*n =* 88), with no statistically significant differences observed, ensuring the reliability of subsequent model development and validation.

### Independent predictors of unfavorable outcomes

Seven predefined variables were forced into the multivariate model. Diabetes mellitus was not statistically significant (OR = 0.96, 95%CI: 0.18–5.03, *p =* 0.958) and was excluded from the final nomogram. The final model included six independent predictors: age, hypertension, GCS score ≤ 8, modified Fisher scale, SIRI, and treatment modality (Table 2).

**Table 2:** Univariate and multivariate analysis of factors associated with poor functional outcome.

Variable	Univariate analysis		Multivariate analysis	
	OR (95% CI)	*P*-value	OR (95% CI)	*P*-value
Age	1.03 (1.01–1.05)	0.015	1.06 (1.02–1.10)	0.003
Female	0.83 (0.49–1.41)	0.502		
Hypertension	2.32 (1.39–3.92)	0.002	2.41 (1.05–5.72)	0.041
Diabetes mellitus	2.05 (0.82–5.26)	0.127		
Smoking	0.85 (0.29–2.32)	0.762		
Alcohol use	0.52 (0.07–2.30)	0.425		
Temperature	1.14 (0.59–2.19)	0.698		
SBP	1.01 (1.00–1.02)	0.101		
DBP	1.01 (1.00–1.03)	0.134		
GCS ≤ 8	14.68 (7.03–33.91)	0.001	4.22 (1.43–13.51)	0.011
Hunt-Hess >2	11.40 (6.31–21.47)	0.001	5.17 (2.05–13.67)	<0.001
Maximum diameter	1.08 (1.02–1.15)	0.021	1.09 (1.00–1.21)	0.088
WBC	1.22 (1.14–1.32)	0.001	0.38 (0.01–5.04)	0.605
NEUT	1.24 (1.16–1.34)	0.001	2.86 (0.22–85.99)	0.571
LYMPH	0.48 (0.28–0.76)	0.003	14.32 (0.76–886.49)	0.212
MONO	9.11 (3.64–24.32)	0.001	0.09 (0.00–9.00)	0.366
HGB	0.99 (0.97–1.00)	0.098		
PLT (zscore)	0.84 (0.64–1.08)	0.189		
ALB	0.95 (0.90–1.00)	0.072		
K	0.86 (0.53–1.37)	0.537		
NA	0.99 (0.97–1.01)	0.448		
INR	46.96 (4.14–603.60)	0.002	3.15 (0.06–177.87)	0.573
APTT	1.04 (1.00–1.08)	0.054		
NLR	1.12 (1.08–1.17)	0.001	1.12 (0.97–1.29)	0.124
PLR (zscore)	1.37 (1.06–1.78)	0.016	2.41 (0.61–9.22)	0.197
LMR	0.63 (0.53–0.75)	0.001	0.76 (0.53–0.95)	0.062
SII (zscore)	1.90 (1.44–2.59)	0.001	0.15 (0.02–1.30)	0.086
SIRI	1.25 (1.16–1.37)	0.001	1.36 (1.04–1.82)	0.033
Treatment method		0.001		0.010
Endo vs. Med	0.12 (0.04–0.30)	0.001	0.17 (0.04–0.61)	0.009
Mic vs. Med	0.2 (0.07–0.50)	0.001	0.44 (0.10–1.76)	0.254
Parent Artery		0.045		0.189
ACom vs. ACA	0.59 (0.18–2.04)	0.389	0.68 (0.13–3.75)	0.653
ICA vs. ACA	1.94 (0.64–6.26)	0.248	2.80 (0.55–16.07)	0.226
MCA vs. ACA	1.23 (0.38–4.24)	0.731	1.51 (0.27–9.25)	0.645
Other vs. ACA	0.89 (0.27–3.07)	0.852	0.79 (0.13–4.84)	0.795
PCom vs. ACA	0.73 (0.22–2.50)	0.603	0.76 (0.14–4.34)	0.755

OR, Odds Ratio; 95% CI, 95% Confidence Interval; SBP, Systolic Blood Pressure; DBP, Diastolic Blood Pressure; GCS, Glasgow Coma Scale; Hunt-Hess, Hunt-Hess Scale; WBC, White Blood Cell Count; NEUT, Neutrophil Count; LYMPH, Lymphocyte Count; MONO, Monocyte Count; HGB, Hemoglobin; PLT, Platelet Count; ALB, Albumin; K, Kalium (Potassium); NA, Natrium; INR, International Normalized Ratio; APTT, Activated Partial Thromboplastin Time; NLR, Neutrophil-to-Lymphocyte Ratio; PLR, Platelet-to-Lymphocyte Ratio; LMR, Lymphocyte-to-Monocyte Ratio; SII, Systemic Immune-Inflammation Index; SIRI, Systemic Inflammation Response Index; Endo, Endovascular Therapy; Med, Medical Management; Mic, Microsurgical Clipping; ACA, Anterior Cerebral Artery; ACom, Anterior Communicating Artery; ICA, Internal Carotid Artery; MCA, Middle Cerebral Artery; PCom, Posterior Communicating Artery.

In the multivariate model, advanced age remained an independent risk factor for unfavorable outcomes (OR = 1.05, 95%CI: 1.01–1.09, *p =* 0.020). Hypertension was an independent predictor (OR = 2.96, 95%CI: 1.26–6.97, *p =* 0.013). Diabetes mellitus showed no significant association (OR = 0.96, 95%CI: 0.18–5.03, *p =* 0.958).

Admission GCS score ≤ 8 was an independent strong predictor (OR = 4.74, 95%CI: 1.50–14.96, *p =* 0.008). The modified Fisher scale showed an overall significant effect (*p =* 0.015). Compared with mFS 1, mFS 4 was associated with a markedly increased risk of unfavorable outcomes (OR = 25.05, 95%CI: 3.47–180.57, *p =* 0.001); mFS 3 showed a significant association (OR = 5.36, 95%CI: 1.02–28.14, *p =* 0.047), while mFS 2 showed a trend without reaching statistical significance (OR = 4.53, 95%CI: 0.91–22.45, *p =* 0.064).

Importantly, elevated admission SIRI was independently associated with an increased risk of 12-month unfavorable outcomes (OR = 1.20, 95%CI: 1.08–1.34, *p =* 0.001).

The overall effect of treatment modality was significant (*p =* 0.039). Compared with conservative medical management, endovascular therapy was associated with a lower risk of unfavorable outcomes (OR = 0.17, 95%CI: 0.04–0.71, *p =* 0.015). Microsurgical clipping did not reach statistical significance compared with medical management (OR = 0.33, 95%CI: 0.08–1.37, *p =* 0.126).

No severe multicollinearity was observed (all VIF < 5), supporting the reliability of the regression model. The Hunt–Hess grade was not included in the model due to collinearity with GCS score, as predefined in the statistical analysis plan.

### Association between SIRI and unfavorable outcomes in aSAH patients

To verify the stability of the association between SIRI and 12-month unfavorable outcomes, sequential multivariate adjusted analyses were performed (Table 3). In the unadjusted model (Model 1), elevated SIRI was significantly associated with an increased risk of unfavorable outcomes (OR = 1.20, 95%CI: 1.08–1.34, *p =* 0.001). After additional adjustment for gender and diabetes mellitus in Model 2, the association of SIRI with unfavorable outcomes remained robust (OR = 1.20, 95%CI: 1.08–1.34, *p =* 0.001). In Model 3, with further adjustment for hemoglobin, APTT, and albumin, SIRI remained an independent predictor (OR = 1.21, 95%CI: 1.08–1.35, *p =* 0.001).

**Table 3:** Multivariate logistic analyses of the association between SIRI and poor functional outcome.

Variable	Model 1, unadjusted		Model 2^a^		Model 3^b^	
	OR (95%CI)	*P*	OR (95%CI)	*P*	OR (95%CI)	*P*
SIRI	1.19 (1.10–1.29)	<0.001	1.19 (1.10–1.29)	<0.001	1.19 (1.10–1.29)	<0.001
Age	1.05 (1.01–1.08)	0.005	1.04 (1.01–1.08)	0.005	1.05 (1.01–1.08)	0.007
GCS ≤ 8	3.57 (1.35–9.43)	0.01	3.68 (1.38–9.80)	0.009	3.66 (1.38–9.74)	0.009
Hunt-Hess>2	5.38 (2.42–11.96)	<0.001	5.35 (2.41–11.88)	<0.001	5.21 (2.26–12.00)	<0.001
Hypertension	2.33 (1.12–4.83)	0.023	2.36 (1.14–4.90)	0.021	2.35 (1.13–4.89)	0.022
Treatment method		0.0013		0.0036		0.0322
Endo vs. Med	0.14 (0.04–0.47)	0.001	0.14 (0.04–0.47)	0.001	0.14 (0.04–0.47)	0.001
Mic vs. Med	0.34 (0.10–1.15)	0.083	0.34 (0.10–1.13)	0.078	0.33 (0.10–1.11)	0.074
Sex (Male)			1.22 (0.58–2.58)	0.598	1.18 (0.51–2.70)	0.696
HGB					1.00 (0.98–1.03)	0.885
APTT					1.01 (0.96–1.06)	0.679
ALB					0.99 (0.91–1.08)	0.907

^a^Adjusted for gender. ^b^Adjusted for gender, hemoglobin, activated partial thromboplastin time, and albumin. OR, Odds Ratio; 95% CI, 95% Confidence Interval; SIRI, Systemic Inflammation Response Index; GCS, Glasgow Coma Scale; Hunt-Hess, Hunt-Hess Scale; Endo, Endovascular Therapy; Med, Medical Management; Mic, Microsurgical Clipping; HGB, Hemoglobin; APTT, Activated Partial Thromboplastin Time; ALB, Albumin.

**Figure 2:**
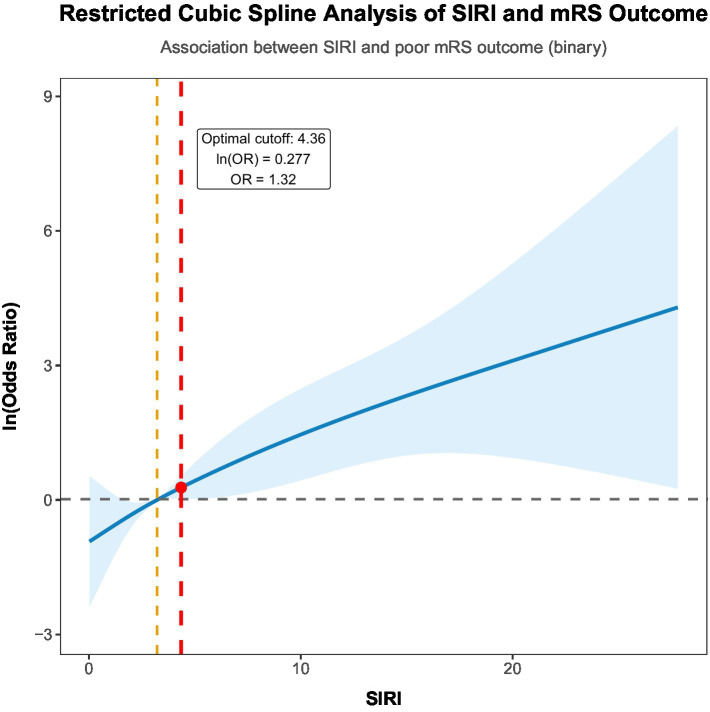
Restricted cubic spline (RCS) plot illustrating the association between the SIRI and log (OR) of unfavorable outcomes (mRS ≥ 3). The solid line denotes the smoothed log (OR) curve, adjusted for age, hypertension, GCS score, modified Fisher scale, and treatment modality. Dashed lines represent the 95% confidence interval (CI), with the reference line anchored at the median SIRI value, and the vertical dotted line indicates the optimal SIRI cutoff value (4.36). Three extreme SIRI values above 30, all belonging to the unfavorable outcome group, were processed by tail truncation to prevent statistical separation in the regression model.

Restricted cubic spline regression was performed to examine the nonlinear relationship between SIRI and the risk of unfavorable outcomes (P for nonlinearity = 0.020; Figure 2). The optimal cutoff value for SIRI was determined to be 4.36. At this threshold, the log-transformed odds ratio was ln(OR) = 0.277, corresponding to an OR = 1.32. A threshold effect was identified: the risk of unfavorable outcomes increased slightly when SIRI was ≤ 4.36, whereas the risk rose steeply when SIRI exceeded 4.36.

### Construction of the prediction model and nomogram visualization

To translate the identified prognostic factors into a clinically actionable tool, we developed a comprehensive prediction model for 12-month unfavorable outcomes in patients with aSAH. The model incorporated six variables identified by multivariate logistic regression: age, hypertension, GCS score ≤ 8, modified Fisher scale, SIRI, and treatment modality.

**Figure 3:**
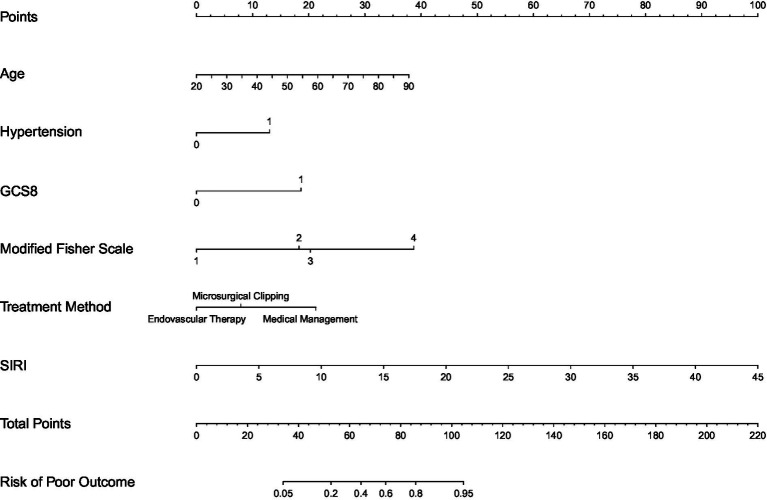
Nomogram for predicting unfavorable outcome risk in aSAH patients, developed based on a multivariate logistic regression model. Individualized predicted probabilities of adverse outcomes are derived by summing the scores of each included variable and mapping the total score to the risk axis.

A nomogram was constructed to visually quantify each predictor’s contribution and enable individualized risk assessment (Figure 3). The practical application of this nomogram follows a straightforward four-step process: First, identify the patient’s specific value for each predictive variable on the corresponding axis. Second, draw a vertical line upward from each variable value to the “Points” axis to obtain the assigned score for that factor. Third, sum the scores of all six variables to calculate the total prognostic score. Finally, draw a vertical line downward from the total points to the “Probability of Unfavorable Outcome” axis to obtain the individualized predicted risk of 12-month poor functional prognosis.

To illustrate its clinical application: consider a 75-year-old patient without a history of hypertension, presenting with an admission GCS score > 8, a modified Fisher scale of 2, an admission SIRI of 4.54, and subsequently treated with endovascular therapy. By summing the assigned points for each of these specific variables on the nomogram, the patient receives a total score of 57. Drawing a vertical line downward from this total score to the risk axis yields an approximate 30% predicted probability of experiencing an unfavorable 12-month functional outcome (Figure 3).

### Model performance evaluation and validation

To evaluate the reliability and clinical applicability of the prognostic model, its discriminative ability, calibration, and clinical utility were systematically assessed in the training cohort (*n =* 170) and temporal validation cohort (*n =* 88).

**Figure 4:**
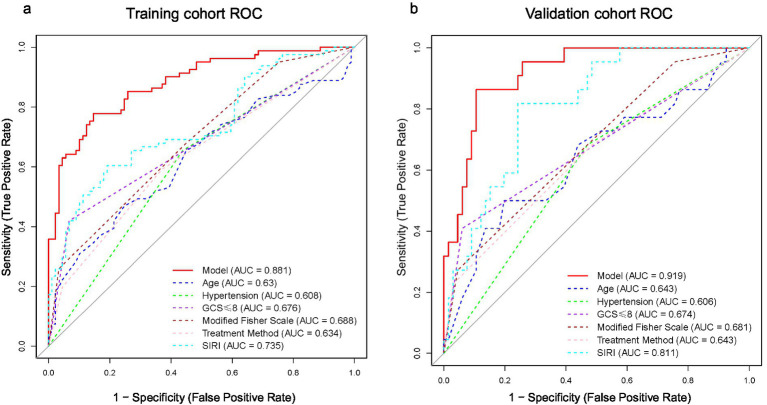
Comparison of ROC curves between the comprehensive model and individual predictors. **(a)** Training cohort; **(b)** validation cohort. The solid curve corresponds to the comprehensive model (training cohort: AUC = 0.881; validation cohort: AUC = 0.919). Among the individual predictors, the SIRI curve had the highest discriminative power (training cohort: AUC = 0.735; validation cohort: AUC = 0.811), and the other curves represent the remaining five core independent prognostic factors.

Receiver operating characteristic (ROC) curve analysis was performed for the 6-variable nomogram, SIRI, and other individual predictors. In the training cohort, the nomogram yielded an AUC of 0.881 (95% CI: 0.83–0.93). In the temporal validation cohort, the AUC remained high at 0.919 (95% CI: 0.86–0.98), indicating excellent discriminative performance. As an individual predictor, SIRI achieved an AUC of 0.735 in the training cohort and 0.811 in the validation cohort, which was higher than that of other single variables, supporting its independent prognostic value (Figure 4).

**Figure 5:**
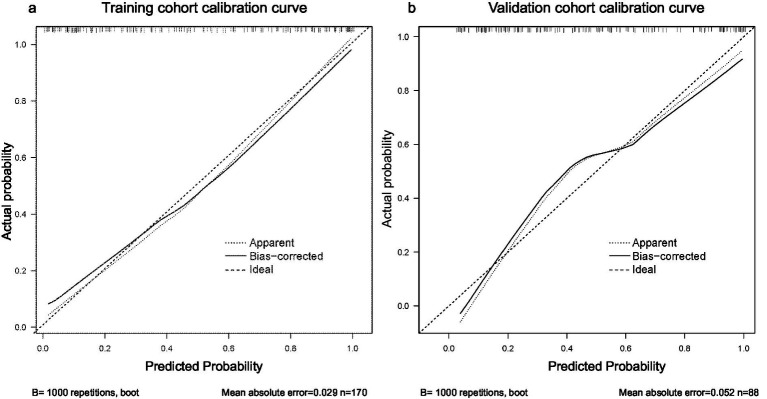
Calibration curves of the prognostic model. **(a)** Training cohort. Mean absolute error (MAE) = 0.029 (sample size *n =* 170); **(b)** Validation cohort. MAE = 0.052 (*n =* 88). The dotted black line represents the apparent calibration curve, the solid black line denotes the bias-corrected (bootstrap) calibration curve, and the dashed black line represents the ideal calibration line. X-axis: Predicted probability; Y-axis: Actual probability.

Calibration curves were plotted to assess consistency between predicted and observed probabilities. In the training cohort, the bootstrap-corrected curve closely approximated the ideal diagonal, with a mean absolute error (MAE) = 0.029 and a non-significant Hosmer–Lemeshow test (*p >* 0.05). In the temporal validation cohort, the calibration curve also showed favorable agreement, with an MAE = 0.052 and a non-significant Hosmer–Lemeshow test (*p >* 0.05), confirming good model calibration (Figure 5).

**Figure 6:**
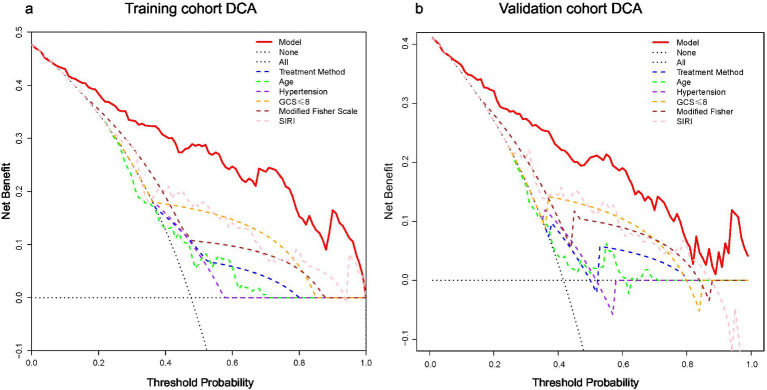
Decision curve analysis (DCA) of the prognostic model: **(a)** Training cohort and **(b)** validation cohort. The *x*-axis is the threshold probability (0–1), and the *y*-axis is the net benefit.

Decision curve analysis (DCA) was used to evaluate clinical net benefit. Within the clinically relevant threshold probability range of 5–50%, the nomogram provided a higher net benefit than the “treat all” or “treat none” strategies, as well as single prognostic indicators including GCS score or modified Fisher scale, supporting its superior clinical utility (Figure 6).

## Discussion

To investigate the association between admission SIRI and unfavorable prognosis (mRS ≥ 3) in patients with aSAH, validate its independent prognostic value, and explore its clinical utility for risk stratification, our study identified three key insights regarding the SIRI’s role in aSAH prognosis: first, admission SIRI independently predicts unfavorable outcomes, with a multivariate OR of 1.20 (95% CI: 1.08–1.34, *p =* 0.001) after adjusting for confounding factors; second, restricted cubic spline analysis reveals a non-linear relationship, with an SIRI >4.36 marking a clear threshold for high adverse outcome risk; and third, the nomogram combining SIRI with standard clinical factors (age, hypertension, GCS score ≤ 8, modified Fisher scale, and treatment modality) delivers strong prognostic performance (AUC = 0.881 in the training cohort and 0.919 in the temporal validation cohort). Together, these results confirm that the SIRI is a dependable inflammatory marker for aSAH prognosis and risk stratification.

aSAH’s poor outcomes largely stem from secondary brain injury, a process driven by interconnected pathological mechanisms (Brandon et al., 2016; Marcey, 2021). When an aneurysm ruptures, blood leaking into the subarachnoid space breaks down, releasing hemoglobin and heme that spark immune activation (Brandon et al., 2016; Marcey, 2021). This causes blood–brain barrier damage, cerebral edema, vasospasm, and neurotoxicity—all worsening neurological harm. Among these processes, systemic inflammation is a linchpin: the tug of war between pro-inflammatory damage and anti-inflammatory repair directly shapes how secondary injury progresses and how well patients recover in the long term (Rowland et al., 2012). This is why inflammatory markers hold promise as prognostic tools in aSAH.

Inflammation sits at the heart of aSAH’s pathophysiology. Rupture triggers a systemic inflammatory response where the innate immune system springs into action: neutrophils and monocytes rush to the injury site, releasing reactive oxygen species, pro-inflammatory cytokines, and extracellular traps that damage blood vessels and neurons (Rowland et al., 2012; Jinman et al., 2025). Simultaneously, lymphocytes—critical for calming inflammation and aiding neurorepair—are suppressed by stress-induced cortisol and cytokine-driven cell death (Rowland et al., 2012). This lopsided response, with too much destructive inflammation and too little repair, becomes a major driver of secondary brain injury and poor outcomes, showing the importance of tracking inflammatory status in aSAH care.

What sets the SIRI apart from other conventional markers is its ability to capture the complete inflammatory picture of aSAH. Unlike single-cell markers (e.g., white blood cell count) or partial ratios (NLR and PLR) that only pick up fragments of inflammation, the SIRI weaves together three key immune cell types: neutrophils and monocytes (the drivers of pro-inflammatory damage) and lymphocytes (the mediators of anti-inflammatory repair) (Aokai et al., 2025). Furthermore, compared to other composite indicators like the systemic immune-inflammation index (SII)—which relies on platelets—SIRI is theoretically more highly tailored to aSAH pathophysiology because it specifically incorporates monocytes. Monocytes are the primary precursors to brain-infiltrating macrophages, which act as the central drivers of erythrocytic clearance, neuroinflammation, and subsequent cerebral vasospasm after hemorrhage (Brandon et al., 2016; Jing et al., 2022). This lets the SIRI measure the dynamic balance between harm and healing—a defining feature of aSAH’s inflammation. By encapsulating this multifaceted dysregulation, the SIRI offers a more complete view of disease progression risk than markers that only scratch the surface.

The SIRI also has practical advantages over existing aSAH prognostic tools in real clinical settings. For one, it is derived from routine blood counts with no room for observer bias, making it objective and consistent unlike subjective scales like the Hunt–Hess grade. It is also widely accessible and affordable: blood tests are available in every clinical setting, with no need for invasive procedures (such as spinal taps for cerebrospinal fluid) or costly imaging (such as CTA for vasospasm checks). It also enables dynamic monitoring: repeated blood draws can track how inflammation changes after treatment or during complications (like vasospasm), allowing clinicians to adjust care in real time. And since it is non-invasive, it is easier on patients and has a lower risk, making it suitable for use throughout treatment.

Moreover, 12-month outcomes in aSAH are profoundly influenced by secondary complications (Marcey, 2021). Our results indicate that patients with unfavorable outcomes had significantly higher incidences of delayed cerebral ischemia (DCI) and cerebral vasospasm. Given that systemic inflammation drives endothelial dysfunction, microthrombosis, and subsequent ischemia (Rowland et al., 2012; Jing et al., 2022), early elevated SIRI likely acts not only as a direct predictor of primary brain injury but also as an upstream marker for these devastating secondary complications. In this context, a high admission SIRI may reflect a hyper-inflammatory cascade that mediates its detrimental effects on long-term outcomes indirectly by predisposing patients to subsequent DCI and vasospasm. Although our current sample size and the single-baseline measurement of SIRI limit the statistical power to perform a robust causal mediation analysis, future large-scale prospective studies incorporating dynamic inflammatory monitoring and mediation modeling are warranted to definitively disentangle these direct and indirect causal pathways.

This study has several limitations. First, our single-center retrospective design may introduce selection bias. Although we performed temporal validation to enhance model reliability, the lack of external validation in independent cohorts still limits the generalizability of these findings to other healthcare settings. Second, SIRI was measured only at a single time point upon admission, capturing merely the hyperacute inflammatory state rather than the dynamic inflammatory trajectory. Early inflammation (days 0–3) differs substantially from delayed inflammation (days 4–14), which is more closely linked to delayed cerebral ischemia and vasospasm. Furthermore, although all blood samples were drawn within 24 h of admission (median: 9.6 h, IQR: 4.3–17.5 h), variability in the exact timing of blood collection post-ictus may still introduce fluctuations in cell counts. Third, unmeasured factors (such as chronic anti-inflammatory use or smoking) may have influenced inflammation and outcomes. Finally, we did not assess the specific molecular links between the SIRI and secondary brain injury, so the biological pathways behind these findings remain incompletely understood.

Future research should address these gaps and build on our findings. Multicenter prospective studies with diverse groups—including older patients, those with comorbidities, and those treated in non-tertiary settings—are needed to confirm the SIRI’s prognostic value and the nomogram’s reliability in real-world care settings. Serial SIRI monitoring studies could clarify how changes in inflammation over time relate to complications such as vasospasm and long-term outcomes. Mechanistic research should explore the specific cells and molecules that connect the SIRI to secondary brain injury. Additionally, interventional trials could test whether targeting inflammation in high-risk patients (SIRI >4.36) improves outcomes, using the SIRI as a quick measure of treatment response. Finally, combining the SIRI with other biomarkers or imaging features might make risk stratification even more precise, leading to more comprehensive tools for aSAH care.

## Conclusion

Admission SIRI is an independent predictor of 12-month unfavorable functional outcomes in patients with aSAH. A threshold of SIRI > 4.36 effectively identifies high-risk individuals, above which the probability of poor prognosis increases steeply. The SIRI-integrated nomogram combines inflammatory status with key clinical and radiological predictors, yielding excellent discrimination and calibration across both training and temporal validation cohorts. This validated tool enables accurate, individualized risk stratification and provides robust evidence to support personalized clinical decision-making for patients with aSAH.

## Data Availability

The data analyzed in this study is subject to the following licenses/restrictions: the datasets presented in this article are not publicly available due to privacy and ethical restrictions, as they contain information that could compromise the confidentiality of patients. Access to the data is restricted to authorized researchers subject to institutional and ethical approvals. Requests to access these datasets should be directed to Lin Y-X, lyx99070@126.com.
